# Molecular Pathology of Retinoblastoma

**DOI:** 10.4103/0974-9233.65498

**Published:** 2010

**Authors:** Mallikarjuna Kandalam, Moutushy Mitra, Krishnakumar Subramanian, Jyotirmay Biswas

**Affiliations:** Department of Ocular Pathology, Vision Research Foundation, Sankara Nethralaya, Chennai, India

**Keywords:** Pathology, Proteomics, Retinoblastoma, Target Therapy

## Abstract

Retinoblastoma (RB) is an embryonic neoplasm of retinal origin. For many years, scientists have sought the fundamental origins of tumorigenesis, with the ultimate hope of discovering a cure. Indeed, these efforts have led to a significant understanding that multiple molecular and genetic aberrations, such as uncontrolled proliferation and the inhibition of apoptosis that contribute to the canonical characteristics of tumor biology. Despite these advances, a thorough understanding, such as the precise cells, which are the targets of neoplastic transformation, especially in solid tumors, is currently lacking. The focus of this review is to emphasize the molecular defects involved in the RB tumor progression and mechanisms associated with inhibition of tumor cell apoptotic processes. This review also discusses the importance of target molecules characterization and their potential therapeutic or prognostic use in RB disease.

## CLINICAL INFORMATION: INCIDENCE AND SURVIVAL RATES

Retinoblastoma (RB) is an embryonic neoplasm of retinal origin in children; with an average incidence of one case for every 15,000–20,000 live births. Approximately, 45% of RB cases are hereditary in nature (15% unilateral and as many as 30% bilateral cases), whereas others are sporadic and present as unilateral tumors.^1^ Approximately 12–15% of patients with RB have a family history, where the tumor phenotype segregates as an autosomal-dominant trait with high (90%) penetrance. Individuals harboring a germline *RB1* gene mutation are predisposed to the development of several other cancers throughout life including bone and soft tissue sarcomas, melanoma, brain tumors and have a 50% risk of transmitting their germline *RB1* gene mutation to offspring.^1^,^2^

Survival rates for RB patients vary widely between developed and developing countries, with figures as high as 90–98% in the United States and Europe and as low as 24% in some African countries.^3^–^8^ This disparity has been attributed to the advanced stage of diagnosis (e.g., orbital RB) in less industrialized nations. For example, tumors occurring in India are advanced by the time children are referred to an ophthalmologist which may be due to diagnostic delay at original presentation prior to referral. Pathologic examination of some enucleated eyes demonstrates features that suggest a risk for future metastatic disease in 15–20% of the cases.^9^,^10^

## TREATMENTS: BENEFITS AND RISKS IN RETINOBLASTOMA

External beam radiotherapy (EBR) was the mainstay of treatment for RB patients until the 1990s. However, the risk of second malignancies from EBR led investigators to seek alternative treatment strategies in patients with hereditary RB.^11^ In the past decade, radiation-sparing therapies that incorporate primary systemic chemotherapy and aggressive focal consolidation with cryotherapy, transpupillary thermotherapy, and brachytherapy have evolved.^12^–^17^ Early recognition of risk factors for metastasis in RB such as diffuse invasion of the choroid, invasion of the post laminar, and surgical end of the optic nerve and orbit, has improved survival rates due to the intense chemotherapy given to these cases. The success of these approaches has been extensively reported and additional benefits observed.^18^ Most notable has been a decrease in the incidence of secondary pineal tumors, suggesting a possible protective effect from the chemotherapy.^18^

## GENOMIC VARIATIONS ASSOCIATED WITH RETINOBLASTOMA

Inactivation of both copies of the *RB1* gene (located at 13q14) in a retinal cell, through mutations or epigenetic modifications, initiates the onset of RB. This event is followed, as in other cancers, by the sequential acquisition of additional genetic abnormalities that define the course leading to tumor formation and metastasis.^19^–^24^ Genomic instability contributes to the progression of retinoma to malignant RB.^25^–^27^ In humans, this progression is characterized by loss of both copies of the *RB1* gene in retinoma followed by changes in the copy number of oncogenes such as *MYCN* (2p24.3), *E2F3* and *DEK* (6p22), KLF14 (7q32), and *MDM4* (1q32) as well as tumor suppressor genes *CDH11* (16q21) and *p75NTR* (17q21). It has also been shown that when *RB1* and *TP53* are inactivated in mice, RB develops.^28^,^29^ Collectively, these observations indicate that beyond biallelic inactivation of *RB1*, additional “hits” are required for the development of RB tumors in humans and mice.^30^–^32^

## MOLECULAR DEFECTS IN RETINOBLASTOMA

### Cell cycle protein abnormalities

*RB1* codes for the RB protein, Rb, which functions as a tumor suppressor oncogene by controlling the cell cycle through complex interactions of multiple kinases and their inhibitors which, together, form the Rb pathway. In the absence of mitogenic stimuli, Rb activity is engaged to inhibit cell-cycle progression through inhibition of transcription of multiple genes required for S-phase entry.^33^–^35^ However, Rb function can be disrupted by the overexpression of D-type cyclins^36^,^37^ or loss of p16^INK4A^ a CDK inhibitor in various cancers.^38^,^39^ Additionally, Rb is the target of the HPV-E7 oncoproteins involved in the etiology of cervical cancer.^40^ Recently, some human papillomavirus (HPV) strains such as HPV 16, 18, 6a, 33, 11, 31, 35, and 51 have been described in fresh tumor tissue from RB patients.^41^–^45^ However, it is unclear whether HPV is the causative agent for RB development. In contrast, an earlier study has shown neither HPV nor any other pRb-inactivating human DNA tumor viruses play a role in the development of RB, regardless of RB1 genotype.^46^ Despite recent advances in the understanding of Rb function, the precise mechanism of RB development remains incompletely understood. Hence due to the continuing controversy regarding the concept of RB development, it is imperative to understand the molecular pathogenesis of malignant transformation and progression of the RB tumor to develop novel and specific therapeutic agents for targeted therapy.

### Theory of cancer stem cells in retinoblastoma

**Hurdles to killing the tumor cells:** For over a century, scientists have fanatically sought the fundamental origins of tumorigenesis, with the ultimate hope of discovering a cure. Indeed, these efforts have led to a significant understanding that multiple molecular and genetic aberrations, such as uncontrolled proliferation and the inhibition of apoptosis, contribute to the canonical characteristics of cancer. Despite these advances in our knowledge, a thorough understanding, such as the precise cells, which are the targets of neoplastic transformation, especially in solid tumors, is currently lacking. An emerging hypothesis is that cancer arises and is sustained from a rare subpopulation of tumor cells with characteristics that are highly similar to stem cells, such as the ability to self-renew and differentiate. In addition, more recent studies indicate that stem cell self-renewal pathways that are active primarily during embryonic development and adult tissue repair may be abnormally activated in various cancers.

A stem cell is defined by its ability to proliferate, self-renew and most importantly, is its ability to retain competence over time. The ability to retain competence is a feature that distinguishes it from progenitor cells in the same tissue. Stem cells have been shown to be expressed in various tissues, and their physiological functions to self-renew enables tissue homeostasis as well as to regenerate new cells after tissue injury. Several studies have also shown the expression and function of stem cells in cancer tissue, and these have been termed cancer stem cells. Seigel *et al*.^47^ observed the presence of a small subpopulation of cancer stem cells (ABCG2 positive) and neural stem cells (MCM2 positive) in tumors from transgenic mice, human RB cell lines, and a small cohort of archival human RBs. ABCG2 is a half ATP-binding cassette (ABC) transporter expressed on plasma membranes. Overexpression of ABCG2 in cell lines confers resistance on a wide variety of anticancer drugs including mitoxantrone, daunorubicin, doxorubicin, topotecan, and epirubicin.^48^–^50^ MCM2 is one of the six members of the family of minichromosome maintenance (MCM) proteins.^51^ MCM proteins are components of the prereplicative complex, which binds to replication origins in the G1 phase of the cell cycle and is essential for the initiation of DNA replication.^52^ MCM2 is a proven marker for detecting neural stem cells.^53^ Since primitive neuroectodermal cells are involved in RB tumorigenesis,^54^ the presence of these neural stem cells could make the tumor more aggressive. Mohan et al.^55^ reported the presence of ABCG2 and MCM2 in a large cohort of RB tumors that correlated significantly with invasive tumors. In addition, a recent study has demonstrated that RB primary tumors harbor cells that express stem cell marker, CD44 and retinal progenitor markers, PROX1 and syntaxin 1A.^56^ The cancer stem cell theory seems to fit in well with some of the yet unexplained concepts of metastasis such as their ability to remain quiescent and be re-activated by secondary factors of the secondary “niche,” to generate a metastatic lesion after a period of dormancy. The presence of cancer stem cells in RBs might have a significant impact on future treatment strategies.

## OXIDATIVE STRESS IN RETINOBLASTOMA

Reactive oxide species such as superoxide radicals (O_2_^•–^), hydroxyl radicals (^•^OH), and hydrogen peroxide (H_2_O_2_) play a key role in the initiation and progression of carcinogenesis.^57^ The cumulative production of reactive oxygen- and reactive nitrogen-species through either endogenous/exogenous insults is termed “oxidative and nitrosative stress.” Nitric oxide is a small messenger molecule that was first discovered as a potent vasodilator known as the endothelium-derived relaxing factor that is produced and released by vascular endothelial cells.^58^ Nitric oxide is synthesized from l-arginine by the action of nitric oxide synthases (NOS).

There are three distinct isoforms of this enzyme, encoded by three different genes. Two of the NOS isoforms are constitutive and calcium-/calmodulin-dependent-the endothelial and neuronal types (eNOS and nNOS, respectively); the third is inducible (iNOS), and is not dependent upon calcium/calmodulin for its enzymatic action.^59^ Recent studies have investigated the expression and the activity of iNOS in human cancer. An increased level of iNOS expression and/or activity has been found in the tumor cells of gynecological malignancies, in the stroma of breast cancer cells,^60^ and in the tumor cells of head and neck cancer.^61^ In the past few years, data regarding the promoting effects of iNOS on tumor development *in vivo* have been mounting. Krishnakumar *et al*.^62^ has demonstrated the expression of eNOS and iNOS in RB tumor tissues. When their expression was compared with invasiveness, eNOS was expressed in both the groups; however, the expression of iNOS and nitrotyrosine were significantly higher in invasive tumors.^62^ Currently, the role of nitric oxide in tumor biology is still poorly understood. The complex biological actions of this ubiquitous signaling molecule necessitate careful experimentation to adequately assess its contribution in RB.

## EXTRACELLULAR MATRIX DEGRADING ENZYMES AND ROLE OF EPITHELIAL CELL ADHESION MOLECULE IN RETINOBLASTOMA

### What is the role of MMPs in RB invasion?

The hallmarks of RB tumor growth are invasion of the vitreous, choroid, optic nerve, orbit, and brain, through blood and, if located anteriorly, RB spreads via the lymphatics to the submandibular region.^63^ These phenomena require degradation of the surrounding extracellular matrix. Degradation of basement membrane by matrix metalloproteinases (MMPs) is one of the most critical steps in various stages of tumor progression, including tumor angiogenesis, tumor growth, and also local invasion and subsequent distant metastasis.^64^ MMP expression is upregulated and correlates with metastatic potential in many tumors.^65^,^66^ The induction of MMP production is, at least in part, mediated by tumor-stromal cell interaction via a tumor cell surface glycoprotein, CD147 also known as extracellular matrix metalloproteinase inducer (EMMPRIN).^67^ The activities of MMPs are regulated by tissue inhibitors of metalloproteinases (TIMPs).^68^ Adithi *et al*.^69^ has demonstrated higher expressions of EMMPRIN, MMP-2 and MMP-9, and TIMP-1 and TIMP-2 in invasive RB tumors. Poorly differentiated tumors showed higher expression of MMP-2 than tumors that were moderately or well-differentiated tumors. The expression of TIMP-1 and TIMP-2 was higher in invasive tumors. With respect to differentiation, there were higher expressions of TIMP1 and TIMP2 in poorly differentiated tumors, as compared to moderately and well-differentiated tumors.

First, MMPs could degrade the extracellular matrix and contribute to the invasiveness in these tumors. The findings of Adithi *et al*. on MMP-2 and MMP-9 expression in RB concur with other studies on the expression of these MMPs in RB.^70^ Examination of the LHßTag murine transgenic RB model showed that MMP-9 was strongly upregulated and MMP-2 was weakly upregulated.^71^ It is clear that in addition to MMP-2 and MMP-9, TIMP-2, TIMP-1, EMMPRIN also play important roles in RB invasion. Their colocalization could further aggravate the invasiveness and contribute to local immunosuppression in the tumor environment. Thus, targeting any one mechanism may be insufficient to reduce tumor invasiveness. Further studies are needed to understand these complex pathways, which contribute to tumor aggressiveness before considering potential therapeutic targets for the treatment or prevention of invasive RB.

### Role of epithelial cell adhesion molecule in RB

Recently, Krishnakumar *et al*. have shown that epithelial cell adhesion molecule (EpCAM) is highly expressed in RB tumors with invasion compared to tumors without invasion.^72^ EpCAM, also known as ESA or EGP40, is a 40-kDa epithelial transmembrane glycoprotein that is encoded by the GA733-2 gene located on the long arm of chromosome 4. It has been found on the basolateral surface of simple, pseudostratified, and transitional epithelia. *In vivo* expression of EpCAM is related to increased epithelial proliferation and has been shown to correlate negatively with cell differentiation. Recently, Krishnakumar’s lab has demonstrated that EpCAM plays a role in increased cellular proliferation of RB cells. Several genes related to cell cycle arrest, apoptosis were increased and genes related to cell proliferation were significantly downregulated in Y79 cells when treated with EpCAM-specific siRNA.^73^ Hence, EpCAM can be considered a potential target molecule in the therapeutic interventions for RB management.

## ROLE OF PROTEIN 53 FAMILY OF PROTEINS IN RETINOBLASTOMA

Protien 53 (p53) controls powerful stress response as it integrates upstream signals from many types of DNA damage and inappropriate oncogenic stimulation, all of which lead to p53 activation and subsequent regulation of genes involved in cell cycle arrest or apoptosis.^74^ Hence, it is not surprising that this is the most frequent site for genetic alterations found in human cancer.^75^ Recently, Laurie *et al*.^76^ demonstrated that the p53 pathway is inactivated through murine double minute-4 [MDMX] amplification in RB and supported the idea that MDMX could be a specific chemotherapeutic target for treating RB. However, the recent discoveries of other p53 family proteins such as p63 and p73 and their multiple isoforms, some of which are p53 agonistic while others antagonistic, and of their p53-independent roles in neurogenesis and stem cell biology^77^,^78^ added new insights and increased the complexity of analyzing p53 function. The truncated isoforms of p73 (Np73) which is a putative antagonizer of p53 function was found to be overexpressed in several cancers.^79^ The first report on the expression of p63 and p73 proteins in RB was reported by Adithi *et al*.^80^ They reported that p63, p73, and their delta isoforms (truncated forms) were expressed in more than 50% of tumor samples. Delta p63 and delta p73 isoforms are known to have p53 pathway suppressive properties. However, further studies are warranted to confirm their initial findings and to explore the cause of expression of these proteins in RB.

## TUMOR BIOMARKERS IN RETINOBLASTOMA:PROGNOSTIC AND THERAPEUTIC USE

**Figure 1:**
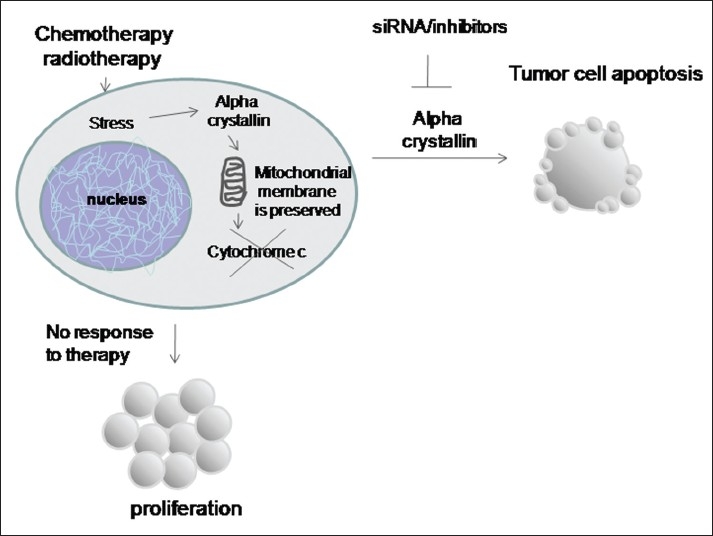
CRYAA expression is induced during oxidative stress in retinal photoreceptors. One of the major roles of alpha crystallin is to preserve the integrity of mitochondria and restrict the release of cytochrome c, subsequently resulting in tumor growth through escape from apoptosis. CRYAA inhibitors or gene silencing methods can enhance the tumor cell apoptotic response to DNA damage

Proteomics is the systematic study of the total proteins expressed in a cell or tissue.^81^ Proteomic analysis is an accurate, sensitive, and high-throughput protein identification strategy.^82^ In the research of the molecular mechanisms of diseases, comparative proteomic analysis has been used as an innovative method to investigate protein expression between cancerous and normal tissues/cells. Understanding the disease progression and identifying the variations in the molecular determinants that possibly drive the tumor progression is mandatory to manipulate these factors for therapy and prognostic evaluations clinically. Mallikarjuna *et al*.^83^ studied the differential protein profile of RB tumors by a combination of two-dimensional gel electrophoresis to separate and visualize proteins and mass spectrometry for protein identification. They identified 27 differentially expressed proteins in RB compared to normal donor retinas. The analysis revealed several deregulated protein changes in RB tumors that play an important role in the metabolic process, cell proliferation, and active transportation process which are the hallmarks of tumor progression. Two of the several proteins upregulated are alpha crystallin A (CRYAA) and peroxiredoxin 6 (PRDX6) which can inhibit apoptotic processes in tumor cells. Another recent study has shown that CRYAA expression was inversely correlated with apoptotic index of RB tumor cells.^84^ It appears that CRYAA may possibly play a role in preventing the apoptosis of tumor cells. One of the major roles of alpha crystallin is to preserve the integrity of mitochondria and restrict the release of cytochrome *c*, subsequently resulting in tumor growth through escape from apoptosis [Figure 1]. Hence, it is imperative to understand the molecular mechanisms involved in CRYAA-mediated prevention of RB cell apoptosis. CRYAA could be an attractive therapeutic target in RB management.

**Figure 2:**
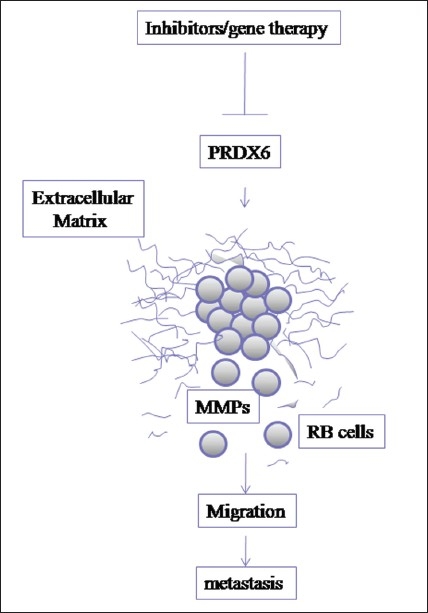
Overexpression of peroxiredoxin 6 leads to invasive phenotype of the tumor cells via upregulation of MMPs which degrade the extracellular matrix proteins and facilitates tumor cells to invade through stroma and leads to metastasis

PRDX6 was significantly upregulated in tumors with invasion compared to tumors without invasion. Earlier, Chang *et al*.^85^ has demonstrated that upregulation of PRDX6 enhanced the *in vitro* proliferation and invasion of breast cancer cells. The enhancement was associated with increasing levels of the urokinase-type plasminogen activator receptor (uPAR), Ets-1 (E26 transformation-specific-1), matrix metalloproteinase (MMP)-9, and RhoC (ras homolog gene family, member C) expression. Higher expression of MMP-2 and 9 were demonstrated earlier in RB tumors by Adithi *et al*.^69^ This could relate to the increased expression of PRDX6, a possible upstream molecule that may induce higher MMP expression in RB. Upon proving this fact in RB, PRDX6 can be considered instead of MMPs for targeted therapy [Figure 2]. The precise role of Prdx-6 is unknown in RB and requires further study. Understanding the precise functional role of these proteins in contributing tumor invasiveness will further help us to target those using novel drugs/inhibitors.

## CONCLUSIONS

Recent studies have significantly contributed in providing a large volume of information about the RB disease mechanism and the pathways of tumors formation or proliferation that help them escape from chemotherapy- or radiotherapy-induced apoptosis. One of the reasons that could account for tumor relapse may be that the RB tumor harbors cancer stem cells. It is well known that cancer stem cells have the characteristics of impaired apoptotic processes. This phenomenon is most commonly associated with p53 mutations or loss of functional wild-type p53 protein and the later has been demonstrated in RB. On the basis of these studies, the goal of maximizing tumor cell destruction with conventional cancer therapy should also include specific cancer stem cell targets through pharmacological inhibition of self-renewal pathways and provoke even a greater apoptotic response by activating the target protein (blocking ΔNp73 to restore p53 function). EpCAM is the functionally evaluated molecule for a role in tumor cell invasion and proliferation. This molecule could be a promising target protein which can be further evaluated in clinical settings for the RB management. Proteomics has contributed significantly in understanding the biology of RB tumors by revealing altered protein expression in tumor cells. The altered proteins belong to different categories such as apoptosis, cell proliferation, signal transduction, metabolic, and active transport processes. PRDX6 and CRYAA could be potential targets in the clinical management of RB. Inhibiting the above target molecules could potentially restore the cell death response in the presence of DNA damage. However, the challenge of pursuing this approach will be to ensure an ample therapeutic manifestation, such that the nearly universal toxicities of chemotherapeutic agents on normal retinal cells are not similarly enhanced.
